# Adolescents’ Perceptions of Contraception Access through Pharmacies

**DOI:** 10.3390/pharmacy8020053

**Published:** 2020-03-28

**Authors:** Ashley H. Meredith, Emily B. Vahary, Tracey A. Wilkinson, Carolyn G. Meagher, Thomas Vielott, Mary A. Ott

**Affiliations:** 1 Department of Pharmacy Practice, Purdue University College of Pharmacy, Indianapolis, IN 46202, USA; vahary@purdue.edu; 2 Department of Pediatrics, Indiana University School of Medicine, Indianapolis, IN 46202, USA; tracwilk@iu.edu; 3 Division of Adolescent Medicine, Indiana University School of Medicine, Indianapolis, IN 46202, USA; cgmeaghe@iu.edu (C.G.M.); tvielott@gmail.com (T.V.); maott@iu.edu (M.A.O.)

**Keywords:** pharmacist, contraception, adolescent, qualitative research

## Abstract

Adolescent pregnancy is an important public health issue, and pharmacist prescribing has the potential to expand contraceptive access and decrease unintended pregnancy. However, little is known about acceptability and uptake of pharmacist prescribing among adolescents, particularly among youth in socially and politically conservative regions of the country. The study objective was to identify how young women in Indiana perceive pharmacist contraceptive prescribing. Participants were recruited from clinics and completed a simulated pharmacist contraception-prescribing encounter; a demographic and behavioral questionnaire; and an in-depth qualitative interview focused on adolescent perspectives on pharmacist prescribing. Data were analyzed using thematic analysis. Sixty young women aged 14–21 years (mean age 17.0 ± 1.7 years) completed in-depth interviews. The majority expressed interest in pharmacist contraceptive prescribing (n = 33, 55.9%). Three overarching themes were identified, focusing on accessibility; quality of care; and pharmacist knowledge and youth friendliness. Subthemes highlighted the need for improved confidential access; a desire for additional pharmacist training in contraception; and interactions with a pharmacist that can relate to the young person. Increased awareness of the perceptions of young people can inform state policies and pharmacy protocols. Pharmacists, because of their accessibility, are well poised and equipped to assist in this public health concern.

## 1. Introduction

Access to contraception is a key strategy to reduce adolescent unintended pregnancy. Pharmacist contraceptive prescribing is an innovative, but under-used, way to increase contraceptive access. Despite national declines in unintended pregnancies, adolescents aged 15–19 have the highest rates, with three out of four pregnancies in this age group unplanned [1]. Adolescents may have limited access to primary care or women’s health services and therefore may be unable to obtain a prescription for contraception [2]. Barriers to accessing contraception are greater the younger the adolescent is and for those with low incomes [2,3,4]. These barriers include lack of affordability or insurance, transportation, privacy concerns, and medical providers that are unwilling or unable to provide contraceptive services [2,3,4]. Difficulty accessing preventative services such as contraception can lead to increased healthcare costs and innovative ways to expand access are needed [4].

With geographic, financial, system, and privacy barriers to obtaining contraception from medical providers, states may be looking toward pharmacists to help fill in these gaps. Pharmacists are uniquely posed to improve contraceptive access due to their prevalence within the community and frequency of use, with more than half of young women visiting a pharmacy at least once per month [5]. Legislation has been passed in several states permitting pharmacists to prescribe contraceptives to women [5,6,7]. Even in states where pharmacy access legislation has passed, there remain significant barriers to adolescent access to care. Of the 11 states that currently allow for pharmacist prescribing of contraceptives, only three of these extend these services to women under 18 years of age [8]. A majority (81%) of patients utilizing pharmacist prescribing in California and Oregon were between the ages of 18 and 34, with only 2% younger than 18 years [9]. Pharmacy prescribing has not significantly improved contraception access for adolescents most at risk for unintended pregnancy. A better understanding of acceptability is necessary to target expansion of pharmacist prescribing to adolescents.

While there are adult data evaluating the acceptability of pharmacist-prescribed contraception, less is known about acceptability and uptake of pharmacist prescribing among adolescents. The majority of adult women aged 18–44 years would utilize pharmacist prescribing if it was provided [3,10]. Of the women who were not currently using a form of contraception, 41% indicated that they would be likely to utilize pharmacist prescribing to obtain contraception, if available [3]. Previous studies have shown that younger women aged 10–25 are interested in pharmacist prescribing of contraception if available [11]. However, many of these studies evaluated the use of emergency contraception as opposed to contraception methods that are utilized more long-term. Additionally, these studies did not examine the perspectives of younger adolescents who may be more dependent upon parents and have additional access barriers to contraception. 

While a vast majority of women are healthy and have no medical contraindications for hormonal contraception, research with adult women who have chronic medical conditions or who take multiple daily medications shows more expressed concerns related to pharmacist prescribing of contraception compared to those without a chronic medical condition [10]. Approximately 20–25% of children and adolescents experience some type of chronic illness [12]. The vast majority of these chronic illnesses are not contraindications to contraceptive use; however, young people with chronic illness may view pharmacy access to contraception differently than those without chronic illness. There is also a need to understand contraceptive acceptability among adolescents with chronic illness.

Youth in socially and politically conservative regions of the country have a decreased ability to consent to reproductive health care, less access to contraception, and experience more social stigma when accessing contraception [13,14,15]. These regions are defined based on various reproductive healthcare metrics, with a focus on adolescent ability to consent to reproductive healthcare, ability to obtain comprehensive sexual education, confidentiality, and a reproductive freedom index [16]. Thus, there is a need to specifically understand the perspectives of adolescents in these areas of the United States, such as the Midwest where this research was conducted. The objective of this study was to identify how adolescents in Indiana perceive pharmacist prescribing of contraception. 

## 2. Materials and Methods 

This study was completed as part of a larger study of adolescent capacity to self-screen and consent to contraception in a pharmacy setting. After approval by the institutional review board of Indiana University with a waiver of parental permission, the larger study recruited 394 female adolescents, with approximately equal numbers from primary care and pediatric subspecialty clinics (i.e., rheumatology, cardiology, headache) to understand the perspectives of both adolescents without, and with, chronic illness. A subset of participants (n = 60) in the larger study completed a simulated pharmacist contraception-prescribing encounter, a questionnaire that included demographics and behaviors, and an in-depth one-on-one qualitative interview [17,18]. The simulated pharmacist contraception-prescribing encounter included a self-screen for medical contraindications and method-specific counseling information based on an expert-created protocol published previously and available at pharmacyaccessforms.org [16]. Participants from both clinic types were recruited via convenience sampling to complete a semi-structured one-on-one qualitative interview, which was conducted in person in a private space at a location of the participant’s choosing (e.g., clinic, coffee shop, library). Interviews were conducted by a trained research assistant and participants received a $20 gift card. The interview guide specifically evaluated participant perspectives on pharmacist prescribing: “If pharmacists in your area were allowed to prescribe birth control, would you choose to have a pharmacist counsel and prescribe one of these methods of birth control? Why or why not?” This was followed by up to eight questions to better understand the young person’s reasoning, such as concerns about getting birth control directly from a pharmacist, or characteristics of pharmacists that would make the adolescent feel comfortable using pharmacist prescribing. Descriptive statistics were used to assess demographic data. Audio recordings were transcribed and deductively analyzed to identify key themes and subthemes related to acceptability and decision-making around pharmacist contraceptive prescribing [19]. Two researchers independently reviewed all transcripts and identified themes. The researchers then met and finalized the themes before independently coding all transcripts. Researchers compared codes and discussed discrepancies until all codes had been reconciled. A third researcher was available to resolve any discrepancies, but was not utilized. Responses were compared between younger and older adolescents (<18 years vs. ≥18 years) and those with and without chronic illness. We found no substantive differences between younger and older adolescents, so we present these data together. We discuss differences between adolescents with and without chronic illness. 

## 3. Results

### 3.1. Participant Demographics

A total of 60 females aged 14–21 years completed in-depth interviews. The average age of participants was 17.0 years (SD ± 1.7 years), with 39 (65%) younger than 18 years and 21 (35%) aged 18 years or older (Table 1). Participants were recruited equally from both primary care and pediatric subspecialty clinics. Fewer than half of participants were sexually experienced, defined as “penis in vagina” (n = 26, 43%), and the majority expressed interest in pharmacist contraceptive prescribing (n = 33, 56%). The only differences between participants 18 years or older and those younger than 18 years were that older participants reported more sexual experience (67% vs. 31%; *p* < 0.05) and more birth control use (71% vs. 31%; *p* < 0.05). The only differences between participants with and without chronic illness were that participants with chronic illness were less likely to report diverse ethnicities (20% vs. 93%, *p* < 0.05), more likely to report private insurance (87% vs. 13%, *p* < 0.05), and more likely to report a medical contraindication to contraception (27% vs. 17%, *p* < 0.10).

### 3.2. Perspectives on Pharmacist Contraceptive Prescribing

Three overarching themes were identified, focusing on accessibility, quality of care and knowledge, and comfort with pharmacist. Subthemes highlighted the need for improved confidential access, a desire for additional pharmacist training, and interactions with a pharmacist that can relate to the young person. Participants from both primary care and subspecialty clinics expressed support for pharmacist prescribing as a method to increase accessibility. Participants younger than 18 years more commonly voiced concerns related to confidentiality and privacy. 

#### 3.2.1. Accessibility

The first theme focused on access to birth control through a pharmacy. Participants felt that obtaining contraception would be more convenient and likely require less time than making an appointment at their provider’s office. Some participants also mentioned having limited transportation to get to doctor’s offices, which tended to be a further drive than a local community pharmacy.

“Honestly it was hard to find because the Planned Parenthood that was in Avon when they got rid of it, it was pretty hard to find another place. Looking online it took me quite a while to find someplace that could do this and cover it with my insurance. So if they ever got rid of the family planning down at Eskenazi, which is already a 30 minute drive, I’d have to find another place and it would be a hassle.”—17 year old, without chronic illness

“Because it’s [pharmacist prescribing] much more convenient. I don’t have to schedule a doctor’s appointment and I could just go across the street and get what I need.”—15 year old, without chronic illness

#### 3.2.2. Quality of Care and Knowledge

The second theme was the knowledge and quality of care provided by the pharmacists. Some participants felt that a pharmacist would possess an increased knowledge about available products than other providers.

“Possibly because I know that for pharmacists their whole job is medicine. So they could possibly be more educated on the medical side of it rather than a doctor.”—17 year old, with chronic illness

“That’s their [pharmacists] area. They [pharmacists] know about medicine and pills and risk... just the effectiveness of it and how to take it if it’s something that you need to take a certain way.”—18 year old, without chronic illness

Other participants expressed concerns related to the care and knowledge base of a pharmacist that would be prescribing contraception. One specific concern that was discussed multiple times was that a community pharmacist may not have access to the patient’s entire medical history and would not be able to make the most informed recommendation. This increases the need to ensure that screening instruments utilized by pharmacists are both thorough and utilize patient-friendly terminology.

“I guess I could rush it and not understand all the risks, or the pharmacist might not know, or maybe leave something out in the health thing, and the pharmacist might not know and prescribe me, not prescribe, but give me some medication that might not go well with my body.”—18 year old, with chronic illness

“Confidentiality, knowledge of medical history and any prior situations that they [pharmacist] may be unaware of... If they signed a confidentiality document and if they were certified to do so. So I guess if they had some extra schooling for it that would make me feel better.”—18 year old, without chronic illness

#### 3.2.3. Comfort with Pharmacist

The final theme that emerged related to characteristics of the prescribing pharmacist that would make the participant feel most comfortable. Overall, participants expressed a preference for a female pharmacist, and one who takes the time to explain details relevant to the contraceptive (e.g., side effects, timing of administration, etc.). Participants also explained that having a younger pharmacist, or someone who would be able to relate to the patient, would be preferred.

“Female because they would understand what you’re going through. It wouldn’t be really awkward talking, it would be awkward talking to a male than a female because they don’t really understand. I think all pharmacists should speak English unless they come across a person with a different language then they should have like pharmacists that speak different languages if they have to like talk to someone that knows a different language. So I really wouldn’t have a problem as long as they speak English to me. If they really communicate and they just don’t like hear like they just don’t make it all about getting your medicine. I would like for a pharmacist to ask me how my day was or how they’re doing or something like that.”—15 year old, without chronic illness

“I’m not sure language or race or anything would matter. Just a welcoming person. I guess just someone that will approach me and say ‘Can I help you with something?’ or just make me feel comfortable and not ignore me. If guess if they are knowledgeable, if I ask a question and they know what to say, and how they say it. I think I’d be able to trust them.”—19 year old, without chronic illness

### 3.3. Participants with and without a Chronic Illness

Half of participants came from primary care (n = 30) and subspecialty care clinics (n = 30). Those from subspecialty care clinics all had at least one chronic illness while those from primary care clinics did not have chronic illness. Similar numbers of participants with and without a chronic illness expressed interest in pharmacist contraception prescribing (n = 16 [53%] vs. n = 17 [59%]). Perspectives expressed by those with and without chronic illness were similar as they related to interest in pharmacy access. Those participants with a chronic illness expressed more concerns about a pharmacist possessing the appropriate knowledge about their medical history or having the ability to tailor birth control method choice to their medical condition(s).

“I like to know all the interactions with the drugs I am taking. Pharmacist(s) may not know my health history... The pharmacist should contact the doctor before prescribing any meds so they know the condition of the patient.” –19 year old, with chronic illness

“As long as they [pharmacist] talked about it to me like you are and go through all of the possibilities and the risks then yes. They would have to have my full medical history as well and confidentiality that I would prefer.”—18 year old, with chronic illness

## 4. Discussion

Adolescents in our study desire an increased access to contraception. Both younger and older participants, as well as those with and without chronic illness, support pharmacist contraceptive prescribing. Specifically related to pharmacist contraceptive prescribing, young people expressed concerns related to confidentiality and privacy in the standard community pharmacy environment. These results are consistent with studies assessing perceptions of pharmacist prescribing in women of other ages [3,10]. The detailed qualitative data obtained through our study can be used to address any particular concerns young women may have with obtaining contraception directly from a pharmacist, to ensure that pharmacist prescribing processes are inclusive for women of all ages. Training programs, implementation materials, and continuing education modules for pharmacists prescribing contraception should continue to address areas of concern for women accessing contraception at the pharmacy regardless of their age, and our findings augment the necessity for that element of implementation. 

While similar numbers of participants with and without chronic illness expressed interest in pharmacist contraception prescribing, those with chronic illness more commonly expressed concerns related to pharmacist knowledge of their past medical history and medical condition(s). Our study participants with chronic illness reported less sexual experience and use of hormonal birth control than those without chronic illness. Additionally, as they were being treated in a subspecialty clinic for a chronic illness, they were more likely to self-report a potential contraindication to hormonal contraception. Within the larger study on adolescent capacity to self-screen, more than 52% of participants had a self-reported or physician-reported potential contraindication to hormonal contraception, defined as a level 3 or 4 condition based on the Centers for Disease Control and Prevention United States Medical Eligibility for Contraception (CDC USMEC) [20,21]. Combined, these factors may have led to the increased concerns expressed related to pharmacist prescribing.

A common perception from our results, and those previously published, include a lack of awareness or understanding of the training pharmacists receive that enable them to provide a service such as contraceptive prescribing [10]. In addition to the training received during their education, states that currently allow pharmacist contraception prescribing have mandated an additional contraceptive-focused educational update prior to offering services [22]. More must be done to ensure that women seeking access to contraception, and the general public, are aware of the education and training required before a pharmacist is able to prescribe contraception. Many participants in our study stated that their comfort with pharmacist prescribing would be increased if specialized training or certification were required, which indicates a misperception related to the way in which pharmacists are trained.

Limitations of this study include a sample of participants from one geographical region that is generally considered conservative and where pharmacy contraception prescribing is not currently available. However, because our results are similar to previously published studies in other geographic areas, we believe they signal common themes that can be used in wider implementation efforts [3,6,9,10]. Financial concerns for adolescents and state-specific laws regarding consent may be important barriers to consider, but were not addressed in our study design. Additionally, our participants were recruited after encounters within a healthcare system, which could mean we did not reach participants struggling to access a healthcare system to begin with and who may be more likely to access contraception at a pharmacy. Finally, given the small sample size and single geographic region of the participants, results may not be generalizable to all adolescents, yet they can be applied to adolescents that may be at a higher risk for unintended pregnancy given socioeconomic and other factors (e.g., primary care, federally qualified health center populations) and those at risk for contraindications to hormonal contraception (e.g., chronic illness, children’s hospitals). 

Combined with prior results, this study strengthens the continued efforts to expand legislation enabling pharmacists to prescribe contraception [10,23]. Increased awareness of the perceptions and specific needs of young people should be incorporated to proposed and existing legislation to allow women younger than 18 years to access contraception via pharmacist prescribing mechanisms [24]. Increased access to contraception for this adolescent population may ultimately decrease unintended pregnancy rates, while reducing the national economic and societal burden of these pregnancies. Pharmacists are well poised and equipped to assist in this public health concern.

## Figures and Tables

**Table 1:** Participant demographics.

Participant Characteristics	Total n = 60	Without Chronic Illness n = 30	With Chronic Illness n = 30
Age, years (mean ± SD)	17 ± 1.7	17.2 ± 1.9	16.8 ± 1.5
Clinic location, n (%)		--	--
Primary care	30 (50%)		
Subspecialty	30 (50%)		
Interest in Pharmacist Prescribing, n (%) ^a^	33 (56%)	17 (59%)	16 (53%)
Race/ethnicity, n (%)			
African American	17 (29%)	16 (55%)	1 (3%)
White	26 (44%)	2 (7%)	24 (80%)
Latino	7 (12%)	7 (24%)	-
Other/mixed	9 (15%)	4 (14%)	5 (17%)
Insurance type, n (%)			
Public	27 (45%)	23 (77%) *	4 (13%) *
Private	30 (50%)	4 (13%)	26 (87%)
None	3 (5%)	3 (10%)	--
Sexual experience, n (%)	26 (43%)	17 (57%)	9 (30%)
Ever used birth control, n (%)			
Pills	20 (33%)	9 (30%)	11 (37%)
Patch	1 (2%)	1 (3%)	0
Ring	0	0	0
Shot	13 (22%)	10 (33%)	3 (10%)
Implant	3 (5%)	2 (6%)	1 (3%)
Hormonal IUD	0	0	0
Non-hormonal IUD	1 (2%)	0	1 (3%)
Emergency contraception	0	0	0
Condoms	22 (37%)	13 (43%)	9 (30%)
Withdrawal	12 (20%)	8 (27%)	4 (13%)
Current use of birth control, n (%)			
Pills	9 (15%)	3 (10%)	6 (20%)
Patch	1 (2%)	1 (3%)	0
Ring	0	0	0
Shot	11 (18%)	8 (27%)	3 (10%)
Implant	2 (3%)	1 (3%)	1 (3%)
Hormonal IUD	0	0	0
Non-hormonal IUD	1 (2%)	0	1 (3%)
Emergency contraception	0	0	0
Condoms	6 (10%)	3 (10%)	3 (10%)
Withdrawal	4 (7%)	2 (6%)	2 (6%)
Potential contraindication to hormonal contraception (patient-reported), n (%)	13 (22%)	5 (17%)	8 (27%)

^a^ n = 59; * *p* < 0.05
